# Comparison of Commercial Negotiated Price and Cash Price Between Physician-Owned Hospitals and Other Hospitals in the Same Hospital Referral Region

**DOI:** 10.1001/jamanetworkopen.2023.19980

**Published:** 2023-06-23

**Authors:** Yang Wang, Elizabeth Plummer, Yuchen Wang, Peter Cram, Ge Bai

**Affiliations:** 1 Johns Hopkins Bloomberg School of Public Health, Baltimore, Maryland; 2 Neeley School of Business, Texas Christian University, Fort Worth; 3 Burnett School of Medicine, Texas Christian University, Fort Worth; 4 John Sealy School of Medicine, University of Texas Medical Branch, Galveston; 5 Department of Medicine, University of Toronto, Toronto, Ontario, Canada; 6 Johns Hopkins Carey Business School, Baltimore, Maryland

## Abstract

This cross-sectional study compares commercial negotiated prices and cash prices between physician-owned hospitals and other hospitals in the same hospital referral region (HRR) using price information available through the Hospital Price Transparency Rule.

## Introduction

Understanding how physicians’ ownership of hospitals affects patients and payers is an important research area.^1^ Prior research^2,3^ has focused on differences in quality between physician-owned hospitals (POHs) and other hospitals. To our knowledge, no research has examined whether POHs have different prices than their competitors. We hypothesized that POHs would have higher prices than their competitors and examined this hypothesis using information available through the Hospital Price Transparency Rule.^4^

## Methods

This study follows Strengthening the Reporting of Observational Studies in Epidemiology (STROBE) reporting guidelines for cross-sectional studies. Institutional review board approval and consent were not sought because no human participants were involved, in accordance with 45 CFR §46. We identified 175 general acute-care POHs from Physician Hospitals of America and confirmed their POH status using website review as of October 1, 2022. We limited our analysis to POHs located in hospital referral regions (HRRs) that contain at least 1 non-POH (nonprofit or for-profit) general acute-care hospital. Hospitals included in the final analysis are plotted in the eFigure in Supplement 1.

We obtained commercial negotiated prices and cash prices as of January 13, 2023.^5,6^ We focused on 8 Centers for Medicare & Medicaid Services–designated shoppable services^4^: spinal injection, physical therapy–therapeutic exercise, magnetic resonance imaging scan of lower spinal canal, computed tomography scan of abdomen and pelvis, comprehensive metabolic panel, blood test-clotting time, and emergency department visit levels 3 and 4. Hospitals’ characteristics in 2020 (most recent year available) were obtained from RAND hospital data, a compiled version of Medicare Cost Reports. Our statistical methods are outlined in the eAppendix in Supplement 1.

## Results

Our final sample includes 156 POHs and 1116 non-POHs located in 78 HRRs. POHs were smaller (mean [SE] number of beds, 55.0 [5.3] vs 162.0 [6.0]; *P* < .001), more profitable (mean [SE] profit margin, 15.2% [1.4%] vs 7.1% [0.9%]; *P* < .001), and more likely to be for-profit (154 POHs [99%] vs 268 non-POHs [24%]), nonteaching (138 POHs [88%] vs 805 non-POHs [72%]), noncritical access (154 POHs [99%] vs 850 non-POHs [76%]), and located in metropolitan areas (147 POHs [94%] vs 707 non-POHs [63%]) than private non-POHs in the same market. POHs served fewer Medicaid patients (mean [SE] proportion of Medicaid discharge, 3.0% [0.5%] vs 7.1% [0.3%]; *P* < .001) and provided less charity care (mean [SE] charity-care-to-expense ratio, 1.3% [0.4%] vs 3.2% [0.1%]; *P* < .001). Most hospitals in the sample disclosed the commercial negotiated price or cash price for at least 1 of the 8 procedures. Nationwide median commercial negotiated prices and cash prices were lower for POHs by 4% to 33% and 5% to 36%, respectively, for 7 of 8 services (Table).

**Table.** Comparison of National Median Commercial Negotiated Price and Median Cash Price^a^

Procedure (*CPT* code)	Commercial negotiated price			Cash price		
	POH, median (IQR), $	Non-POH, median (IQR), $	Difference, %^b^	POH, median (IQR), $	Non-POH, median (IQR), $	Difference, %^b^
Spinal injection (62323)	1039 (788-1850)	1386 (1094-1844)	−25	1172 (745-1766)	1232 (829-1694)	−5
Physical therapy, therapeutic exercise (97110)	63 (45-96)	90 (72-106)	−30	74 (44-107)	83 (65-113)	−11
MRI of lower spinal canal (72148)	989 (429-1425)	1478 (1054-1778)	−33	1113 (451-1985)	1713 (1208-2035)	−35
CT of abdomen and pelvis (74177)	1265 (572-2408)	1580 (1015-2561)	−20	1628 (600-2942)	2531 (1622-3397)	−36
Comprehensive metabolic panel (80053)	80 (18-140)	72 (35-123)	11	88 (33-232)	127 (81-197)	−31
Blood test, clotting time (85610)	25 (7-46)	26 (14-46)	−4	36 (11-65)	39 (26-56)	−8
ED visit level 3 (99283)	481 (351-605)	588 (434-803)	−18	414 (290-595)	456 (356-635)	−9
ED visit level 4 (99284)	734 (588-949)	927 (714-1191)	−21	764 (457-1127)	756 (573-1021)	1

Abbreviations: *CPT*, *Current Procedural Terminology*; CT, computed tomography; ED, emergency department; MRI, magnetic resonance imaging; POH, physician-owned hospital.

^a^ A total of 112 POHs (72%) and 917 non-POHs (82%) disclosed the commercial negotiated price for at least 1 of the 8 procedures. A total of 108 POHs (69%) and 892 non-POHs (80%) disclosed the cash price for at least 1 of the 8 procedures. National median (IQR) values of each hospital’s median commercial negotiated price among its contracting plans and national median values of each hospital’s cash price are presented.

^b^ Differences were calculated as the difference between median prices divided by the median price for non-POHs.

HRR-level regression results showed that median commercial negotiated prices and cash prices among POHs were 33.7% and 32.7% lower than those of non-POHs, respectively, for the same procedure in the same HRR. Hospital plan–level regression results indicated that POH status was associated with 17.5% and 46.7% lower negotiated prices and cash prices, respectively, for the same procedure and in the same HRR (Figure).

**Figure.**
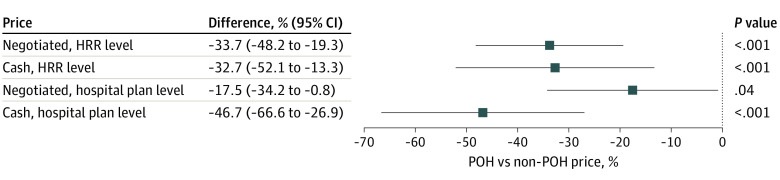
Regression Estimates of Physician-Owned Hospitals’ (POHs) Commercial Negotiated Prices and Cash Prices vs Non-POHs’ Prices in the Same Hospital Referral Region (HRR) For HRR-level analysis, an observation for the negotiated price model is defined as the median value of each hospital’s median commercial negotiated price for a given procedure in an HRR, by POH status (1017 observations); an observation for the cash price model is defined as the median value of each hospital’s cash price for a given procedure in an HRR, by POH status (1007 observations). For hospital plan–level analysis, an observation for the negotiated price model is defined as a commercial negotiated price for a given procedure negotiated between a hospital and a health plan (163 005 observations); an observation for the cash price model is defined as a hospital’s cash price for a given procedure (8801 observations). Additional details of the statistical analysis can be found in the eAppendix in Supplement 1.

## Discussion

This cross-sectional study found that nationwide median commercial negotiated prices and cash prices were lower for general acute-care POHs than for non-POHs in the same market for most common hospital procedures. POHs served fewer Medicaid patients and provided less charity care, which might enable them to accept lower commercial prices (these factors were controlled for in the regression models). This study is limited by its focus on pricing for 8 procedures in 78 HRRs, possible sample-selection biases from omitting hospitals with no available pricing data, the lagged measurement period for hospital characteristics, and the lack of pricing trend information to examine the effect of the Hospital Price Transparency Rule.

## Notes

Supplement 1. **eFigure.** Location of Physician-Owned Hospitals **eAppendix.** Statistical Methods

Supplement 2.
Data Sharing Statement
