# Supplementary material for: Comparison of Commercial Negotiated Price and Cash Price Between Physician-Owned Hospitals and Other Hospitals in the Same Hospital Referral Region
Source: JAMA Netw Open. 2023 Jun 23;6(6):e2319980. doi: 10.1001/jamanetworkopen.2023.19980 (PMC10290252; doi:10.1001/jamanetworkopen.2023.19980)
Supplement: Supplement 1. — eFigure. Location of Physician-Owned Hospitals eAppendix. Statistical Methods [file jamanetwopen-e2319980-s001.pdf]

Supplemental Online Content

Wang Y, Plummer E, Wang Y, Cram P, Bai G. Comparison of commercial negotiated price and cash price between physician-owned hospitals and other hospitals in the same hospital referral region. *JAMA Netw Open*. 2023;6(6):e2319980. doi:10.1001/jamanetworkopen.2023.19980

eFigure. Location of Physician-Owned Hospitals

eAppendix. Statistical Methods

This supplemental material has been provided by the authors to give readers additional information about their work.

© 2023 Wang Y et al. *JAMA Network Open.*

eFigure: Location of Physician-Owned Hospitals

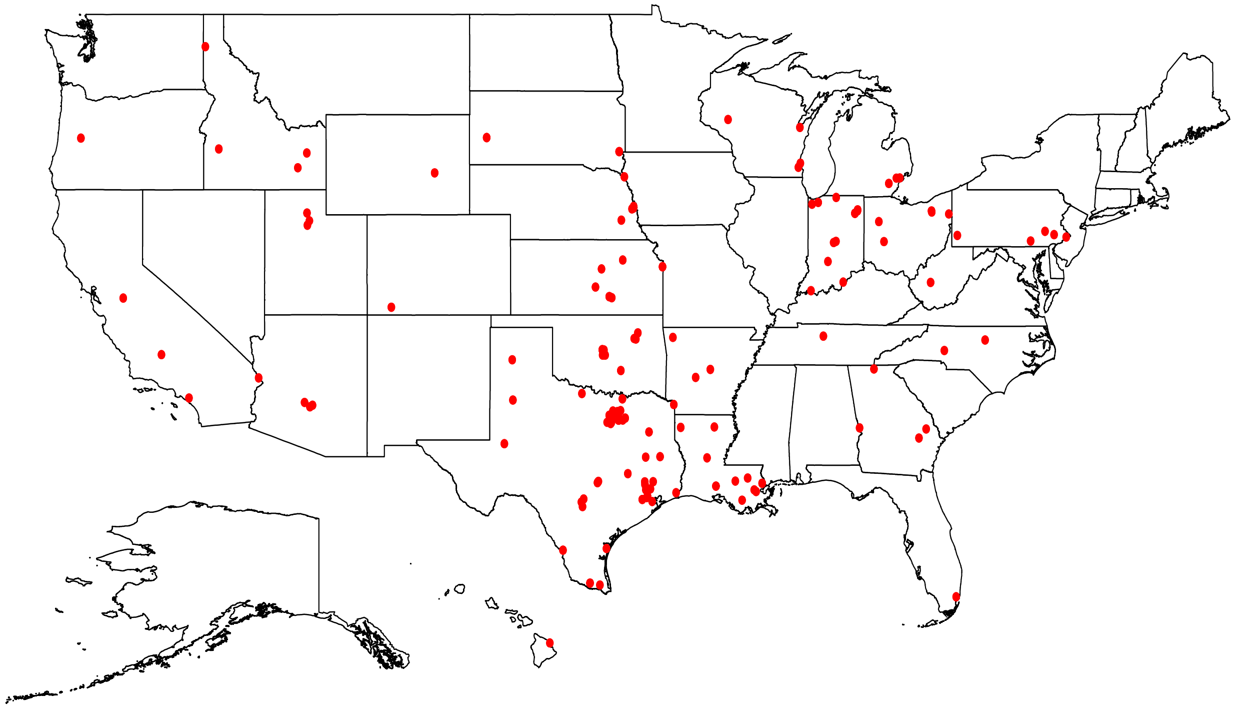

The locations of 156 physician-owned hospitals were plotted.

eAppendix. Statistical Methods

We first compared hospital characteristics, commercial negotiated prices, and cash prices between POHs and non-POHs. Two-sided *t* tests were used to compare means (significance level, $P < .05$). We estimated HRR-level regression models to understand the difference in percentage between median POH prices and median non-POH prices in the same HRR, where prices were log-transformed to enable interpreting results as percentage differences. We also estimated hospital plan–level regression models to examine the association of POH status with price after controlling for hospital characteristics, including ownership type, metropolitan area status, critical access hospital (CAH) status, teaching hospital status, bed size, overall margin (overall net income divided by total revenue), % Medicare patient discharges, % Medicaid patient discharge, and charity-care-to-expense ratio. Both models included procedure and HRR fixed-effects to control for interprocedure and inter-HRR price variations. All standard errors were clustered at the HRR level. Statistical analysis was conducted using STATA statistical software version 14.0 (StataCorp).

© 2023 Wang Y et al. *JAMA Network Open.*
