# Supplementary material for: Comparison of Commercial Negotiated Price and Cash Price Between Physician-Owned Hospitals and Other Hospitals in the Same Hospital Referral Region
Source: JAMA Netw Open. 2023 Jun 23;6(6):e2319980. doi: 10.1001/jamanetworkopen.2023.19980 (PMC10290252; doi:10.1001/jamanetworkopen.2023.19980)
Supplement: Supplement 2. — Data Sharing Statement [file jamanetwopen-e2319980-s002.pdf]

Data Sharing Statement

Wang. Comparison of Commercial Negotiated Price and Cash Price Between Physician-Owned Hospitals and Other Hospitals in the Same Hospital Referral Region. *JAMA Netw Open*. Published June 23, 2023. doi:10.1001/jamanetworkopen.2023.19980

Data
Data available: No

Additional Information
Explanation for why data not available: The data used in this study is publicly available.
